# Biological Fate of Fe_3_O_4_ Core-Shell Mesoporous Silica Nanoparticles Depending on Particle Surface Chemistry

**DOI:** 10.3390/nano7070162

**Published:** 2017-06-30

**Authors:** Estelle Rascol, Morgane Daurat, Afitz Da Silva, Marie Maynadier, Christophe Dorandeu, Clarence Charnay, Marcel Garcia, Joséphine Lai-Kee-Him, Patrick Bron, Mélanie Auffan, Wei Liu, Bernard Angeletti, Jean-Marie Devoisselle, Yannick Guari, Magali Gary-Bobo, Joël Chopineau

**Affiliations:** 1 Institut Charles Gerhardt de Montpellier (ICGM), Montpellier University-Campus Triolet (CNRS UMR 5253/UM/ENSCM), Place Eugène Bataillon, CEDEX 5, 34095 Montpellier, France; estelle.rascol.c2i12@gmail.com (E.R.); christophe.dorandeu@umontpellier.fr (C.D.); clarence.charnay@umontpellier.fr (C.C.); jm.devoisselle@univ-montp1.fr (J.-M.D.); yannick.guari@umontpellier.fr (Y.G.); 2 NanoMedSyn, 15 Avenue Charles Flahault, 34093 Montpellier, France; morgane.daurat2@gmail.com (M.D.); afitz@hotmail.fr (A.D.S.); m.maynadier@nanomedsyn.com (M.M.); 3 IBMM, CNRS UMR 5247/UM/ENSCM Faculty of de Pharmaceutical Sciences of Montpellier 15, Avenue Charles Flahault, CEDEX 05, 34093 Montpellier, France; magali.gary-bobo@inserm.fr; 4 Center of Structural Biochemistry (CNRS UMR 5048/INSERM U 1054/UM), 29 rue de Navacelles, 34090 Montpellier, France; josephine.laikeehim@cbs.cnrs.fr (J.L.-K.-H.); patrick.bron@cbs.cnrs.fr (P.B.); 5 Aix-Marseille Université, CNRS, IRD, Coll de France, CEREGE, 13001 Aix en Provence, France; auffan@cerege.fr (M.A.); wei.yanzi.liu@gmail.com (W.L.); angeletti@cerege.fr (B.A.); 6 Université de Nîmes, Rue Georges Salan, Nîmes 30000, France

**Keywords:** nanoparticles, surface coating, cell-membrane interactions, biodistribution, safety

## Abstract

The biological fate of nanoparticles (NPs) for biomedical applications is highly dependent of their size and charge, their aggregation state and their surface chemistry. The chemical composition of the NPs surface influences their stability in biological fluids, their interaction with proteins, and their attraction to the cell membranes. In this work, core-shell magnetic mesoporous silica nanoparticles (Fe_3_O_4_@MSN), that are considered as potential theranostic candidates, are coated with polyethylene glycol (PEG) or 1,2-dimyristoyl-sn-glycero-3-phosphocholine (DMPC) lipid bilayer. Their biological fate is studied in comparison to the native NPs. The physicochemical properties of these three types of NPs and their suspension behavior in different media are investigated. The attraction to a membrane model is also evaluated using a supported lipid bilayer. The surface composition of NPs strongly influences their dispersion in biological fluids mimics, protein binding and their interaction with cell membrane. While none of these types of NPs is found to be toxic on mice four days after intravenous injection of a dose of 40 mg kg^−1^ of NPs, their surface coating nature influences the in vivo biodistribution. Importantly, NP coated with DMPC exhibit a strong accumulation in liver and a very low accumulation in lung in comparison with nude or PEG ones.

## 1. Introduction

In the past two decades, nanoparticles (NPs) for medical applications have been investigated by numerous researchers. The NPs based theranostic agents combine, in a unique formulation, tracking, imaging, diagnosis, cell-targeting and drug delivery properties. In this emerging field of nanomedicine, Mesoporous Silica Nanoparticles (MSN) are considered as a promising platform for drug-delivery and cell targeting [1]. However, the major obstacle for NPs’ accumulation at the site of interest is the rapid opsonization of the NPs by the liver and spleen macrophages. This opsonization is due to the formation of a protein corona at the surface of the NPs when they enter in contact with biological media [2]. The composition of the corona is particularly dependent on the NPs composition, surface charge and hydrophobicity [3]. Moreover, the protein corona influences the toxicity of the NPs [4]. Different proteins adsorb on native MSN [5], on liposomes prepared with various lipid compositions [6] and in the presence of polymeric groups [7,8,9,10,11]. Grafting of polyethylene glycol (PEG) groups on the NPs is well-known to induce a stealth effect of the NPs [12]. The NPs coated with PEG groups present a longer circulation time, and escape the reticuloendothelial system (RES) capture [13]. The PEG coating brings hydrophilicity at the surface of NPs. Water molecules interact via hydrogen bonds with the PEG moieties, enhancing the hydrodynamic diameter of the NPs [12]. However, this type of grafting induces aggregation in high saline concentration solution [14]. In fact, we previously demonstrated that MSN needed a precise amount of PEG in surface in order to avoid aggregation of particles. If the quantity was not optimized, PEG grafting did not improve colloidal stability [15]. Another strategy to produce a stealth effect, less investigated than the PEG coating, is to coat the surface of NPs with a lipid bilayer. The lipid bilayer, with an average thickness of 5 nm, does not change drastically the hydrodynamic diameter of the NPs [16]. Moreover, the lipid bilayer coating brings new functionalities to the NPs. Indeed, thermally controlled release can be performed by alternating magnetic field or phototherapy [17]. The biocompatibility of lipid coated NPs was generally tested using pegylated lipids [18]. The presence of a magnetic core add interesting functionalities to MSN [19]. The magnetic properties of Fe_3_O_4_ nanocrystals are useful to follow NPs using magnetic resonance imaging (MRI) [20] and produce thermally-controlled drug delivery or hyperthermia treatment [21]. In our previous study, the synthesis and functionalization of magnetic core shell MSN (Fe_3_O_4_@MSN) had been optimized [22]. The results, obtained using the classical MTT assay, showed that 1,2-dimyristoyl-sn-glycero-3-phosphocholine (DMPC) and PEG coated Fe_3_O_4_@MSN were less cytotoxic than the native NPs [22]. Moreover, an early effect of DMPC Fe_3_O_4_@MSN on cells has been observed using real-time dependent impedance measurements translating the difference in cell morphologies after NPs treatments. 

These results supposed that the different physicochemical properties of PEG and lipid coated MSN influence the kinetics of their interaction with cells. Indeed, it was reported that the stability of NPs in biological media has a direct impact on interactions of NPs with cells [23]. To date, the relationships between physicochemical properties and biological effects of NPs are not well understood [24,25]. It has been shown that membrane models can be useful to understand the role of the physicochemical properties of NPs with their toxic effects [26]. Moreover, the behavior and effects of NPs on cell-membrane can be studied using membrane models [27] in well-defined conditions, as previously done for lipid coated silica NPs [5]. The quartz crystal microbalance with dissipation monitoring (QCM-D) is a well suited technique to follow the formation of membrane models, such as supported lipid bilayers (SLB) [28]. This technique provides estimation, in real-time, of a mass or a thickness adsorbed to the surface associated with the viscoelastic properties [29]. The deposition of the NPs, and the kinetics, on different surfaces have been studied using QCM-D as the analytical technique [10,30,31]. The characterization of the interactions between NPs and a supported lipid bilayer (SLB) has been performed to investigate the interaction of various types of NPs in different conditions [32,33,34,35]. However, to the best of our knowledge, the comparison of native, PEG and lipid coated MSN on a membrane model has not yet been studied. Plus, the relationship of physicochemical properties of these three different NPs surfaces in regard to cell internalization and in vivo biodistribution has not been compared before. The goal of this study was to understand the relation between surface chemistry, physicochemical properties, cell membrane interactions, in vivo biodistribution and toxic effects of NPs.

## 2. Results

### 2.1. Synthesis and Characterization of Fe_3_O_4_@MSN 

**Figure 1:**
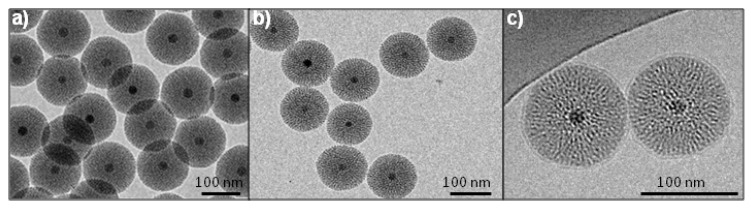
TEM and cryoTEM images of the nanoparticles (NPs); (**a**) Native magnetic mesoporous silica core-shell nanoparticles (Fe_3_O_4_@MSN); (**b**) polyethylene glycol (PEG) Fe_3_O_4_@MSN; (**c**) 1,2-dimyristoyl-sn-glycero-3phosphocholine (DMPC) Fe_3_O_4_@MSN observed by cryoTEM.

Fe_3_O_4_@MSN core shell NPs were prepared by sol-gel reaction, following the optimized protocol previously described [22]. Fe_3_O_4_@MSN NPs present a magnetic core of 18 nm and a mesoporous silica shell of ca. 40 nm radius (Figure 1). Particles with an average primary diameter of ca. 100 nm were grafted in-situ by the addition of PEG_2000_ groups. The primary diameter of PEG Fe_3_O_4_@MSN NPs observed on TEM images was the same than for the native ones. The coating with DMPC lipid bilayers was performed after the synthesis, washing and drying of the native NPs. TEM images allow for the measurement of the primary diameter and the observation of the shape of the NPs (Figure 1a,b), while cryoTEM images revealed also the presence of DMPC lipid coating on Fe_3_O_4_@MSN (Figure 1c). 

**Figure 2:**
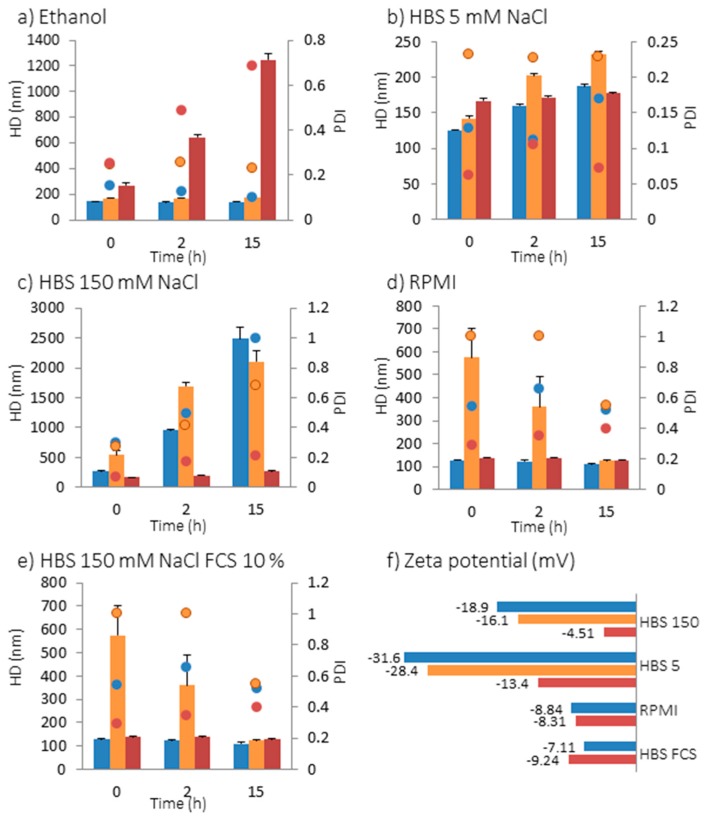
Hydrodynamic diameter (HD) represented by bars and polydispersity index (PDI) represented as dots of native (blue), PEG (orange) and DMPC (red) Fe_3_O_4_@MSN in different media: (**a**) in ethanol; (**b**) in HBS 5 mM NaCl, pH 7.4; (**c**) in HBS 150 mM NaCl, pH 7.4; (**d**) in RPMI cell culture medium (10% fetal calf serum (FCS), pH 7.4) and (**e**) in HBS 150 mM NaCl (pH 7.4) containing 10% FCS; (**f**) Zeta potential in HBS 150, HBS 5, RPMI and HBS 150 mM NaCl containing 10% FCS.

CryoTEM images revealed that the lipid bilayers measured 5 nm in thickness, so the primary diameter of DMPC Fe_3_O_4_@MSN is 10 nm higher than the native and PEG Fe_3_O_4_@MSN. The dispersion and stability of the NPs in suspension in different media were investigated by the measurement of the hydrodynamic diameter using Nano ZS apparatus (Figure 2). The NPs’ surface chemistry influences their dispersion and stability when they were suspended in ethanol, HBS 5 (Hepes buffer 20 mM pH = 7.4 containing 5 mM NaCl), HBS 150 (Hepes buffer 20 mM pH = 7.4 containing 150 mM NaCl), complete cell culture medium Roswell Park Memorial Institute RPMI, and HBS 150 containing fetal calf serum (FCS) 10% (Figure 2). In ethanol, native and PEG Fe_3_O_4_@MSN present a hydrodynamic diameter (HD) of 144.5 ± 2.7 nm and 169.5 ± 0.9 nm, respectively (Figure 2a). These diameters are higher than the primary diameters observed by TEM, and the NPs stayed none aggregated in suspension after 2 and 15 h. Differently, DMPC Fe_3_O_4_@MSN suspended in ethanol presented a HD of 269.9 ± 15.9 nm, which increased to 643.5 ± 19.9 nm after 2 h and 1246 ± 48.2 nm after 15 h. The increase of HD of lipid coated Fe_3_O_4_@MSN in ethanol may be explained by a partial solubilization of the lipids in this solvent. In HBS 5, native Fe_3_O_4_@MSN presents a HD of 125.5 ± 1.2 nm at 0 h, 159.9 ± 1.9 nm after 2 h and 187.5 ± 3.3 nm after 15 h. PEG Fe_3_O_4_@MSN presents a HD of 141.8 ± 3.9 nm after 0 h, 202.1 ± 2.8 nm after 2 h and 232.5 ± 3.1 nm after 15 h. DMPC Fe_3_O_4_@MSN presents a HD of 167.1 ± 3.2 nm after 0 h, 171.6 ± 2.0 nm after 2 h and 177.1 ± 2.3 nm after 15 h. Thus, in HBS 5, native, PEG-grafted and lipid-coated NPs remained individually dispersed during 15 h after initial dispersion (Figure 2b). In HBS 150 buffer, native and PEG Fe_3_O_4_@MSN formed aggregates larger than 1 µm 2 h after initial dispersion (Figure 2c). However, coating with DMPC lipid bilayers was found to stabilize NPs suspension in buffer with high ionic strength (HBS 150). In this buffer, DMPC Fe_3_O_4_@MSN presented a HD of 171.6 ± 0.1 nm after 0 h, 198.0 ± 0.2 nm after 2 h, and 270.9 ± 0.2 nm after 12 h. In cell culture medium (RPMI containing 10% FCS) and HBS 150 containing 10% FCS, lipid coated DMPC Fe_3_O_4_@MSN were found always stable 15 h after dispersion (Figure 2d,e). The good dispersion of lipid coated DMPC Fe_3_O_4_@MSN in HBS 150 buffer and HBS 150 containing 10% FCS was also observed on cryoTEM images (data not shown). In RPMI and HBS 150 containing 10% FCS, native NPs rapidly stabilized, presenting a HD of 128.2 ± 3.0 nm and 110.8 ± 2.0 nm at 0 h, in RPMI and HBS 150 10% FCS, respectively. However, the stabilization of PEG Fe_3_O_4_@MSN in protein-containing media took more time, presenting a HD close to the primary diameter only after 15 h. The zeta potentials (ZP) were measured in all media, except in ethanol (Figure 2f). Native Fe_3_O_4_@MSN presented a ZP of −18.9 ± 1.4 mV and −31.6 ± 0.8 mV, in HBS 150 and HBS 5, respectively. A decrease (in absolute value) of zeta potential to −16.1 ± 3.0 mV in HBS 150 and −28.4 ± 2.6 mV in HBS 5 by grafting by PEG groups was observed, and to −4.51 ± 1.1 mV in HBS 150 and −13.4 ± 0.3 mV in HBS 5, by coating by DMPC lipid bilayers. However, in protein-containing media, the zeta potential remained the same for native and lipid coated Fe_3_O_4_@MSN. The stabilization of native Fe_3_O_4_@MSN in RPMI and HBS 150 10% FCS is potentially due to the formation of a protein corona [23]. The stabilization of PEG Fe_3_O_4_@MSN in protein-containing media was longer due to the stealth effect of the PEG groups at the NPs surface impairing protein adsorption to the NPs surface.

To conclude, the presence of a strong ionic strength or proteins did not modify the physicochemical behavior of DMPC Fe_3_O_4_@MSN, which are therefore the only ones to remain dispersed and stable in biological media. 

### 2.2. Interaction of NPs with Model Membranes Depending of the Surface Coating 

#### 2.2.1. Changes at the Interface between NPs and a Supported Lipid Bilayer 

**Figure 3:**
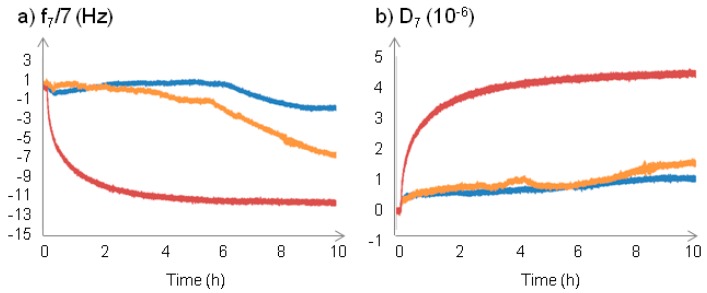
Quartz crystal microbalance with dissipation (QCM-D) sensorgrams following the interaction between NPs and egg phosphatidyl choline (EPC) supported lipid bilayer (SLB). Native (blue), PEG (orange) and DMPC (red) Fe_3_O_4_@MSN were flowed into HBS 150 mM NaCl 10% SCF medium on the top of EPC SLB, at a concentration of 0.25 mg mL^−1^ of NPs. After Fe_3_O_4_@MSN addition in the medium on the top of the EPC SLB during 15 min, the flow was stopped for 10 h. The results on the variations of frequency (**a**) and of the dissipation (**b**) are presented after offset of the lipid bilayer formation.

The measurement of the interactions between NPs and a supported membrane model has been performed using a quartz crystal microbalance with dissipation monitoring (QCM-D). A SiO_2_ coated 5 MHz quartz crystal was used. After injection of EPC (egg phosphatidyl choline) SUV (small unilamellar vesicles) suspension, a SLB (supported lipid bilayer) was formed, presenting a frequency shift of −26 Hz and a dissipation shift less than 0.5 × 10^−6^; typically recorded for a supported bilayer [28]. The surface coated with the SLB was considered as the reference surface, and frequency and dissipation shifts induced by NPs deposition were measured from this surface. NPs were put in contact by flowing a NPs suspension on the top of the SLB in HBS 150 10% FCS medium (Figure 3). In this protein-containing medium, only very little NPs deposition on the lipid bilayer surface for PEG-Fe_3_O_4_@MSN and nativeFe_3_O_4_@MSN was observed. DMPC-Fe_3_O_4_@MSN interaction with the SLB surface resulted in more important frequency and dissipation shifts than those obtained for PEG-Fe_3_O_4_@MSN and native-Fe_3_O_4_@MSN. The kinetics of deposition were very different depending of the surface coating. The deposition of DMPC-Fe_3_O_4_@MSN occurred rapidly during the NPs flowing, inducing a frequency shift of −7.55 ± 1.49 Hz and a dissipation shift of 3.37 ± 0.71 × 10^−6^ after 1 h deposition. The maximum frequency shift observed was −9.81 ± 3.11 Hz and the maximum dissipation shift was 4.46 ± 0.43 × 10^−6^ after 10 h deposition. PEG-Fe_3_O_4_@MSN began to deposit at 4 h 07 ± 27 min, with a maximum frequency shift of −6.88 ± 0.75 Hz and a maximum dissipation shift of 1.49 ± 0.30 × 10^−6^ was obtained after 10 h deposition. NativeFe_3_O_4_@MSN began to deposit at 6 h 16 ± 33 min, with a maximum frequency shift of −3.69 ± 3.03 Hz and a maximum dissipation shift of 0.84 ± 0.30 × 10^−6^ was obtained after 10 h deposition. The deposition rates of the NPs were dependent of the aggregation state and the presence of a protein corona. DMPC-Fe_3_O_4_@MSN, which were well-dispersed and not influenced by the presence of proteins (Figure 2), were found to faster deposit on the SLB (Figure 3). PEG-Fe_3_O_4_@MSN were first aggregated and slowly dispersed due to the presence of proteins (Figure 2), allowing the deposition 4 h 07 ± 27 min after injection in the QCM-D cell (Figure 3). Native-Fe_3_O_4_@MSN stabilized by the presence of the protein corona at their surface (Figure 2), were very well dispersed, so these NPs began to deposit 6 h 16 ± 33 min (Figure 3). 

#### 2.2.2. Time-Dependent Internalization of NPs in Hep-G2 cells 

**Figure 4:**
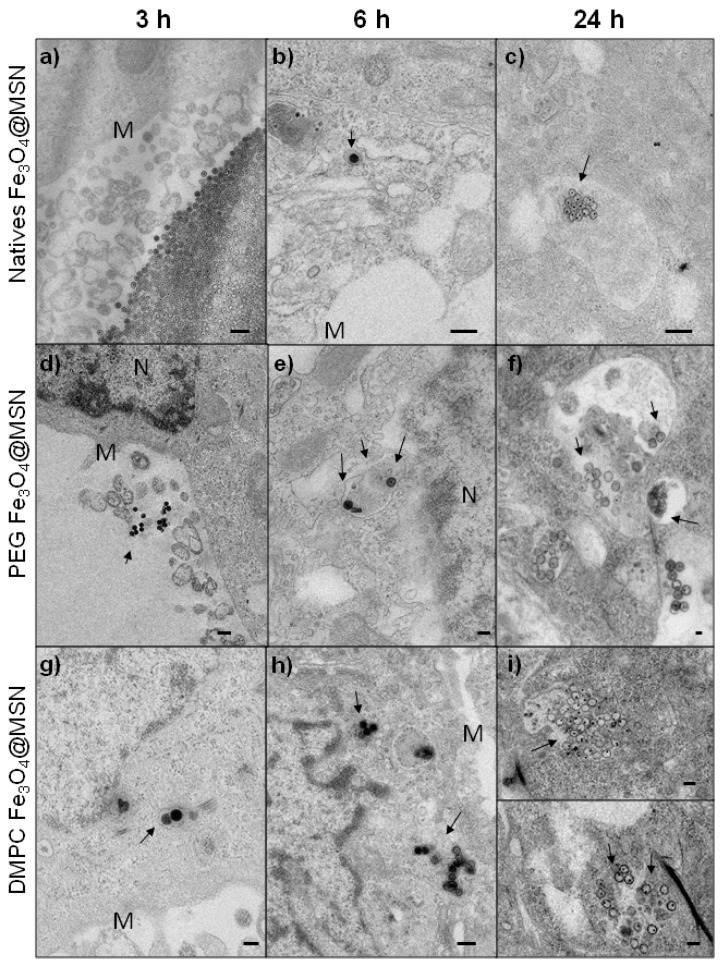
TEM imaging of Hep-G2 cells exposed for 3, 6, and 24 h at 50 µg mL^−1^ for native (**a**–**c**), PEG (**d**–**f**) or DMPC Fe_3_O_4_@MSN (**g**–**i**). The NPs are localized by arrows, near the cell membrane (M) or the nucleus (N).

The uptake of native, PEG and DMPC Fe_3_O_4_@MSN by Hep-G2 cells (human hepatocyte carcinoma) after different incubation times were followed by TEM from ultrathin sections (Figure 4). After 3 h incubation period, native Fe_3_O_4_@MSN were observed aggregated near the cell membrane (Figure 4a). PEG Fe_3_O_4_@MSN were less aggregated than the native Fe_3_O_4_@MSN but were not observed in the cells (Figure 4d). 

Conversely, DMPC Fe_3_O_4_@MSN were observed inside Hep-G2 cells after 3 h of exposure (Figure 4g). After 6 h incubation period, native Fe_3_O_4_@MSN were observed individually in vesicular structures in the cytoplasm (Figure 4b) and some PEG Fe_3_O_4_@MSN were internalized also (Figure 4e). DMPC Fe_3_O_4_@MSN were observed as small groups of NPs in vesicular structures (Figure 4h). These small groups were then observed for natives, PEG and DMPC Fe_3_O_4_@MSN after 24 h of NPs exposure with Hep-G2 cells (Figure 4c,f,i, respectively). This suggests a faster internalization of DMPC Fe_3_O_4_@MSN in human hepatic cells. 

### 2.3. In Vivo Experiments 

#### 2.3.1. In Vivo Biocompatibility 

**Figure 5:**
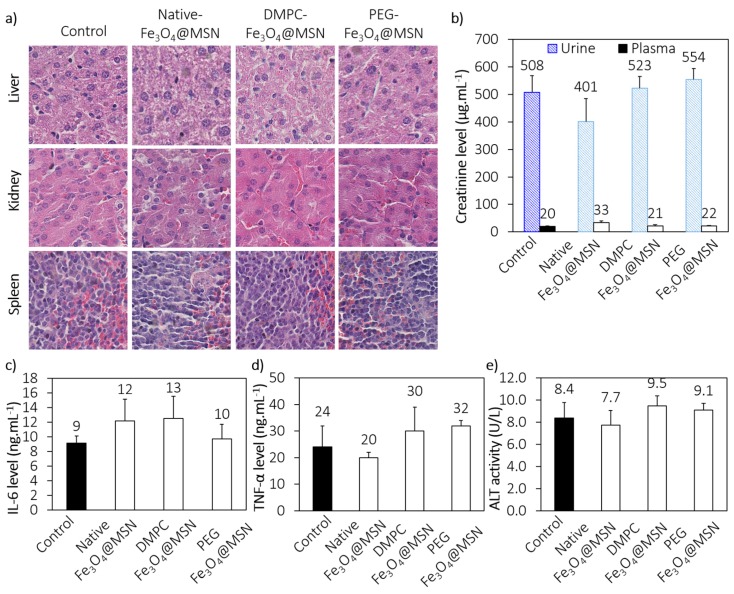
Preliminary toxicological assessment. (**a**) Hematoxylin-eosin sections from paraffin-embedded tissues (kidney, liver and spleen) of control and treated mice; (**b**) Plasma and urine levels of renal biomarker (creatinine); (**c**,**d**) Plasma levels of systemic inflammation biomarkers (IL-6 and TNF-α); (**e**) Plasma level of liver biomarker (ALT).

To estimate the biocompatibility of MSN, mice were injected intravenously with nanoparticles at a concentration of 40 mg kg^−1^. This concentration is high and corresponds to concentration already used to evaluate the acute toxicity of NPs in mice [36]. Four days after the injection of NPs, mice were sacrificed and organs, urines and blood were collected. Histological analyses were performed. No noticeable structural modifications on liver, kidney and spleen were observed (Figure 5a). Moreover, as shown in Figure 5b–e, no significant differences between control and treated mice for the biomarkers such as creatinine (kidney), interleukine-6 (IL-6) and tumor necrosis factor alpha (TNF-α) (systemic inflammation), and alanine aminotransferase (ALT) (liver) were observed, confirming the functional integrity of organs. These results demonstrated the biocompatibility of the nanoparticles under consideration in this work. 

#### 2.3.2. In Vivo Biodistribution 

**Figure 6:**
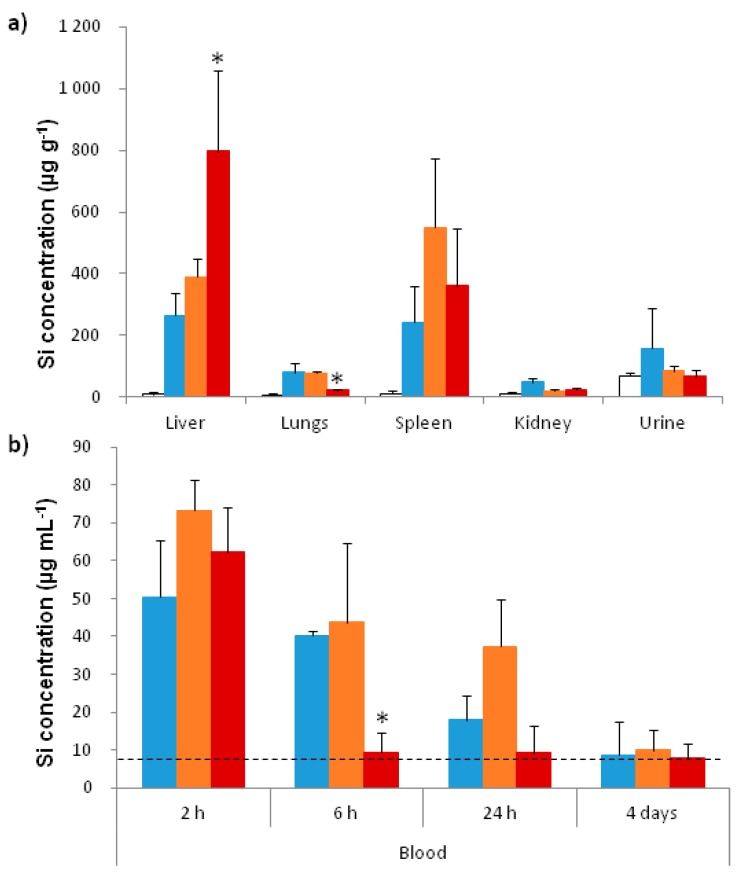
Biodistribution of Fe_3_O_4_@MSN in mice. (**a**) Quantification of silicium in different organs 4 days after injection. Inductively coupled plasma-mass spectrometry (ICPMS) was used after acid digestion to quantify the silicium in the liver, the lungs, the spleen, the kidneys, and urine 4 days after intravenous injection of native (blue), PEG (orange) and DMPC (red) Fe_3_O_4_@MSN at a concentration of 40 mg kg^−1^ in comparison to control mice (white); (**b**) NPs level in blood. The silicium was quantified in the blood 2 h, 6 h, 24 h, and 4 days after intravenous injection of native (blue), PEG (orange) and DMPC (red) Fe_3_O_4_@MSN at a concentration of 40 mg kg^−1^. The dashed line indicates the silicium level found in the blood of control mice. For this experiment 20 mice were divided in 4 groups of 5 animals. Values of histograms represent mean ± SD of values of each animal of a group. * *p* < 0.05 statistically different from all other groups treated with NPs.

The amount of silicium has been quantified using inductively coupled plasma-mass spectrometry (ICP-MS) analysis. No significant elevation of silicium amount was detected in the kidneys and urines 4 days after injection. The quantities of silicium in the blood, the liver, the spleen, and the lungs were different depending of the NPs coating. These differences suggested changes in biodistribution and in pharmacokinetics of the NPs, due to the presence of the coating. Silicium amount in the liver 4 days after injection of DMPC Fe_3_O_4_@MSN is elevated compared to the level in liver of mice treated by native and PEG Fe_3_O_4_@MSN (Figure 6a). This shows a strong capture of DMPC Fe_3_O_4_@MSN by the liver. However the accumulation in the spleen is almost similar between the 3 batches of MSN. Importantly, the lung is a biological barrier that is crucial to avoid and we can see that the amount of silicium in the lung is as low as the background level when mice were treated with DMPC Fe_3_O_4_@MSN. In contrast, this level is higher after injection of native and PEG Fe_3_O_4_@MSN. 

In the blood, 2 h after injection, the levels of silicium are equally elevated between the different batches (Figure 6b). In contrast, 6 h after injection, we can see that the amount of circulating nanoparticles for natives and PEG Fe_3_O_4_@MSN decrease slowly, while DMPC Fe_3_O_4_@MSN do not circulate anymore (Figure 6b). This could be correlated to a rapid and strong uptake by the liver. We note that 24 h after the injection, PEG Fe_3_O_4_@MSN are still well present in blood, suggesting a better circulation time due to the coating.

## 3. Discussion

The presented data demonstrate that native, DMPC and PEG Fe_3_O_4_@MSN stay dispersed and stable during 15 h when diluted in a medium having a low ionic strength (HBS 5 mM NaCl, pH 7.4). When the ionic strength was increased to a more physiological amount of NaCl (HBS 150 mM NaCl, pH 7.4), native and PEG Fe_3_O_4_@MSN rapidly aggregated. The aggregation may be explained by a compression of the electrical double layer [23]. The coating with PEG is supposed to reduce the aggregation of NPs in suspension, by adding a steric repulsion between the NPs surfaces [37]. If the polymer grafting is heterogeneous, there is low electrostatic repulsion between the NPs, and the proteins are adsorbed on the non-coated surfaces leading to a progressive dispersion of the NPs. So the stabilizing effect of polymer grafting is not always obtained, depending of the polymer chain length, of the grafting density and the conformation of the chains at the NPs surface [6]. These different parameters influence the formation of a water shell [38] and the adhesion of some proteins at the surface [39]. Native Fe_3_O_4_@MSN rapidly dispersed in the presence of proteins (SVF 10%, in HBS 150 mM NaCl, pH 7.4 or RPMI). The electrostatic repulsion between negatively charged NPs surface and the proteins is reduced by the high ionic strength [40]. The protein corona forms a stabilizing shell around native Fe_3_O_4_@MSN. On the contrary, PEG Fe_3_O_4_@MSN are aggregated when diluted in HBS 150 mM NaCl, pH 7.4, in the presence or in the absence of proteins, or in RPMI containing SVF 10% and then slowly dispersed in the presence of proteins. The stealth effect expected by NPs coating with PEG is due to a significant reduction of the protein corona [41]. Yet, some proteins are still adsorbed because, as it has been previously described, the adsorption of proteins is a prerequisite to induce a stealth effect [8]. In our study, the adsorption of proteins onto the PEG Fe_3_O_4_@MSN surface was progressive, inducing a reduction of the aggregation state in suspension. DMPC Fe_3_O_4_@MSN stay stable in suspension during 15 h in HBS 150 mM NaCl, pH 7.4 or in cell culture medium (RPMI), containing or not SVF 10% proteins. The reduction of aggregation state of NPs by coating with a lipid bilayer was previously described [42]. The influence of proteins on the aggregation state of NPs has consequences on their interaction with the cell membrane, and potentially on their toxicity [4]. From QCM-D experiments performed in HBS 150 mM NaCl, pH 7.4, containing FCS 10%, DMPC-Fe_3_O_4_@MSN were found to rapidly deposit during the NPs flowing. Conversely, native and PEG Fe_3_O_4_@MSN were found to deposit more slowly. The stabilization of native Fe_3_O_4_@MSN and less importantly PEG Fe_3_O_4_@MSN by the presence of proteins in the medium seemed to reduce the deposition of the NPs on the membrane surface. On the contrary, the DMPC Fe_3_O_4_@MSN stability in suspension was not influenced by the presence of the proteins, and they were rapidly deposited on the membrane model. 

The internalization of DMPC Fe_3_O_4_@MSN by Hep-G2 cells was also faster than the internalization observed for native and PEG Fe_3_O_4_@MSN. DMPC Fe_3_O_4_@MSN were observed inside intracellular vesicles after 3 h while native and PEG Fe_3_O_4_@MSN were observed inside cells after 6 h of incubation. These results suggest an early interaction between DMPC Fe_3_O_4_@MSN and Hep-G2 cell membrane than for the two other types of NPs. This is in accordance with the results obtained by real-time cell impedance measurements reported previously [22]. Using this indirect technique, an early variation of the cell index was observed when the Hep-G2 cell line was exposed to DMPC Fe_3_O_4_@MSN in comparison to native and PEG Fe_3_O_4_@MSN. To conclude on the impact of proteins on NPs cell membrane interactions, the more protein corona is important, the less NPs interact with the cellular membrane. Moreover, native Fe_3_O_4_@MSN, rapidly coated with serum proteins were slowly internalized by Hep-G2 cells, but remained the more cytotoxic according to the MTT assay and real-time cell impedance measurements. In this previous work, native Fe_3_O_4_@MSN induced 40% of cell death at a concentration of 100 µg mL^−1^, when in the same conditions NPs recovered by PEG or DMPC induced 5% or 30% of cell death, respectively. 

From in vivo experiments using mice, no toxic effect was observed four days after intravenous injection of native, PEG, or DMPC Fe_3_O_4_@MSN for a particle concentration of 40 mg kg^−1^ on the renal or hepatic function, neither on inflammation factors. The histological observations show no difference between the liver, the spleen or kidneys of treated and non-treated mice. These results are in accordance with other published works on native MSN [19,36].

However, different biodistribution profiles were observed depending of the particle coating. Firstly, the DMPC Fe_3_O_4_@MSN were quickly cleared from the blood circulation, because no more silicium was detected in comparison to control mice 6 h after injection of the NPs. This could be due, at least in part, to an efficient targeting of the liver. In addition, a large amount of in vivo data indicates that nanoparticles have the capacity to exert adverse pulmonary effects after different ways of exposure and systemic also, and it is a real challenge to avoid lung penetration [43]. Here, the very low level of silicium in the lungs of mice treated with DMPC Fe_3_O_4_@MSN translates the poor accumulation of these NPs in lungs, which is of major interest for a medical application.

Conversely, the amount of silicium 24 h after injection is not significantly different to 6 h for PEG Fe_3_O_4_@MSN, suggesting that the PEG coated NPs are slowly distributed in the organs. The presence of native and PEG Fe_3_O_4_@MSN in the lung may be due to the easier formation of aggregates than with DMPC Fe_3_O_4_@MSN. The prolonged circulation time observed for PEG Fe_3_O_4_@MSN is associated to a poor and slow cell internalization of the NPs. On the contrary, DMPC Fe_3_O_4_@MSN are rapidly internalized by cells; plus the absence of aggregation of DMPC Fe_3_O_4_@MSN at high ionic strength or in the presence of proteins, is a considerable advantage in the challenge of a translational objective. This is the first study on well-characterized monodisperse core-shell MSN comparing native or coated with PEG polymer or DMPC lipid bilayer by in vitro, in vivo experiments, and using cell membrane models. 

## 4. Materials and Methods 

### 4.1. Materials 

All reagents were commercially available and used without any further purification. Hydrated iron oxide [FeO(OH), catalyst grade 30–50 mesh], oleic acid (90%), oleylamine (99%), diethylether (≥99.9%), anhydrous ethanol (≥99.8%), anhydrous pentane (99+%), anhydrous chloroform (99+%), tetraethoxysilane (TEOS, ≥99.9%), cetyltrimethylammonium bromide (CTAB), ammonium nitrate (NH_4_NO_3_), dimethyl sulfoxide (DMSO) and *N*-(2-hydroxyethyl)piperazine-*N*′-[2-ethanesulfonic acid] (Hepes), sodium dodecyl sulfate (SDS) were purchased from Sigma-Aldrich (Saint-Louis, MO, USA). Ultrapure Normatom^®^ acids for trace metal analysis HNO_3_ 67–69% and HCl 34% were obtained from VWR (Atlanta, GA, USA). PlasmaPure H_2_O_2_ 30% was obtained from SCP Science (Québec, QC, Canada). Sodium hydroxide (NaOH) and *n*-docosane (99%) were purchased from Acros (ThermoFisher Scientific, Waltham, MA, USA). Potassium chloride (KCl) was purchased from Prolabo (VWR, Atlanta, GA, USA). Hellmanex was purchased from Hellma (Müllheim, Germany). 1,2-dimyristoyl-sn-glycero-3-phosphocholine (DMPC) and l-α-phosphatidylcholine (EggPC) were purchased from Avanti polar lipids (Alabaster, AL, USA). DPBS buffer (KCl 2.66 mM, KH_2_PO_4_ 1.47 mM, NaCl 137.93 mM, Na_2_HPO_4_-7H_2_O 8.05 mM, pH = 7.4) was provided by Gibco (ThermoFisher scientific, Waltham, MA, USA). Silanized PEG (CH_3_O-PEG_2000_-Si(OCH_3_)_3_) was purchased from Rapp polymer (Tuebingen, Germany).

Concerning cryo-electron microscopy, three microliters of suspension were applied to glow discharged Quantifoil R 2/2 grids (Quantifoil Micro Tools GmbH, Jena, Germany) or Lacey grid (Ted Pella Inc., Redding, CA, USA), blotted for 1s and then flash frozen in liquid ethane using a CP3 cryo-plunge (Gatan Inc., Pleasanton, CA, USA). Before freezing, the humidity rate was stabilized at about 95%. Cryo-EM was carried out on a JEOL 2200FS FEG operating at 200 kV under low-dose conditions (total dose of 20 electrons/Å^2^) in the zero-energy-loss mode with a slit width of 20 eV. Images were taken at a nominal magnification of 50,000× corresponding to a calibrated magnification of 45,591× with defocus ranging from 1.4 to 2.5 μm. 

### 4.2. Synthesis and Characterization of NPs 

The native, PEG-grafted and lipid-coated Fe_3_O_4_@MSN were synthesized following the procedure recently described [22]. The same characterization methods were used. Hydrodynamic diameters and zeta potentials were determined using a Nano ZS apparatus (Malvern Instruments, Malvern, UK). Data were collected from the He-Ne laser light source (λ = 633 nm) at 173° from the transmitted light beam. Stock suspensions of the Fe_3_O_4_@MSN were prepared in stable conditions at a concentration of 10 mg mL^−1^. Pristines Fe_3_O_4_@MSN were dispersed in ethanol 95%, DMPC Fe_3_O_4_@MSN in HBS 150 mM NaCl and PEG Fe_3_O_4_@MSN in HBS 5 mM NaCl. The different suspensions were then diluted in different media at a concentration of 50 µg mL^−1^. The dispersion behavior of the different Fe_3_O_4_@MSN was compared in 5 different media: ethanol, HBS 5 mM NaCl, HBS 150 mM NaCl, HBS 150 mM NaCl containing 10% FCS and complete RPMI (FCS 10%). The same suspensions were analyzed directly after dispersion, 2 h and 15 h later. Results are presented as Z-average obtained in intensity mode, associated to the polydispersity index (PDI).

### 4.3. Interaction of NPs with Membrane Models 

The interactions of Fe_3_O_4_@MSN with model membranes were investigated using a QCM-D E1 setup (Biolin Scientific, Västra Frölunda, Sweden). The system is composed of a measurement chamber containing a 5 MHz quartz crystal sensor with a silica-coated surface. After each measurement, the chamber, the flow module, and the quartz were rinsed with 2 mL 2% Hellmanex and with 5 mL MilliQ water at a flow of 0.5 mL min^−1^. The silica-coated quartz sensor was stored in a SDS 2% solution between two analyses. All measurements were performed at 25 °C. Firstly, the silica-coated surface was rinsed with MilliQ water, dried and placed during 15 min in UV-ozone chamber. Then, HBS 150 mM NaCl was flushed at 100 µL min^−1^, and the quartz sensor was equilibrated for each overtone (1st to 13th). The baseline was obtained using HBS 150 mM NaCl both for the frequency and dissipation recordings. A supported lipid bilayer (SLB) is formed on a silica-coated surface of the QCM-D sensor after fusion of EPC small vesicles SUVs). For SUVs formation, a EPC lipid film was resuspended in Hepes 150 mM NaCl, ultrasonicated using a microtip (Digital Sonifier 250^®^, Branson Ultrasonics Corporation, Danbury, CT, USA), and then centrifuged at 20,000× *g* for 15 min (to remove titanium particles). The supernatant containing SUVs was collected and filtrated through 50 nm porous polycarbonate membrane before coating the SiO_2_ QCM-D sensor. The SLB was formed by adding Egg PC SUVs (50 nm) in HBS 150 mM NaCl buffer at a lipid concentration of 0.1 mg mL^−1^ using a flow of 100 µL min^−1^ for 10 min. The formation of a continuous lipid bilayer is characterized by a frequency shift ∆*f* of −26 Hz and a dissipation shift ∆*D* < 0.5 × 10^−6^ followed on the 7th overtone. The membrane was washed during 1 h with HBS 150 mM NaCl under a flow of 100 µL min^−1^ for stabilization. Before adding NPs on top of the SLB, the NPs dispersant media was injected to recording ∆*f* and ∆*D* changes due to the solvent composition. The NPs were dispersed at 0.25 mg mL^−1^ in HBS 150 mM NaCl containing 10% FCS, and then added on the membrane for 15 min (100 µL min^−1^, 1.5 mL), and the flow was stopped for 10 h. Results were presented after offset of the SLB frequency and dissipation recordings, in order to analyze only the NPs effect. 

### 4.4. Cellular Assays

#### 4.4.1. Cell Culture

The Human hepatocyte carcinoma (Hep-G2) cell line was obtained from Sigma-Aldrich. Cells were cultured in RPMI 1640 (Invitrogen, Carlsbad, CA, USA) supplemented with 10% fetal calf serum (FCS) and 1% penicillin/streptomycin (100 U mL^−1^, 100 µg mL^−1^) and incubated in a cell incubator at 37 °C and 5% CO_2_. Cells were used between passages 20 to 40. Cells were passed once a week and the medium was changed twice a week, keeping cells confluence below 80%. 

#### 4.4.2. Internalization Assay 

Hep-G2 cells were seeded on glass coverslips for 24 h. After controlling their adherence and growing, cells were exposed to 50 µg mL^−1^ of NPs in RPMI for 3, 6 or 24 h at 37 °C and 5% CO_2_. The medium was removed and cells were rinsed twice with DPBS. Cells were fixed by incubation with 2.5% (*v*/*v*) glutaraldehyde in DPBS buffer, for 1 h at room temperature (RT). Then, cells were extensively washed with DPBS. The staining of samples was obtained upon incubation with 1% osmium tetroxide. Samples were dehydrated by ascending grades of EtOH; for impregnation, the samples were firstly treated with a mix EtOH/EPON^TM^ resin (1:1, *v*/*v*) for 1 h, and twice in EPON for 2 h. The polymerization was performed by embedding cells in EPON resin for 12 h at 60 °C, plunged in liquid nitrogen at −195 °C to detach the coverslip, and placed for two days at 60 °C for completing polymerization. The ultrathin sections (70 nm) were obtained using an ultramicrotome (Leica Ultracut, Wetzlar, Germany) and disposed on the copper grids. The grids were incubated in uranyl acetate for 2 min, rinsed in water, and then incubated in lead citrate for 2 min, and finally rinsed with water. For each condition (native, PEG and DMPC Fe_3_O_4_@MSN at 3, 6 or 24 h exposure time of Hep-G2 cells), almost 20 cells were observed, and then the observations were focused on the areas were NPs were observed. 

### 4.5. Biodistribution in Mice 

#### 4.5.1. Animals

Female C57BL/6 mice (25 g) used for this study were procured from Charles River (Wilmington, MA, USA) and housed in the Institutional animal house under standard environmental conditions (23 ± 1 °C, 55 ± 5% humidity and 12/12 h, light/dark cycles) and maintained with free access to standard diet and water. To establish the preliminary toxicological analyses, 4 groups of 5 mice were injected intravenously with 200 µL of saline solution with or without MSN at a concentration of 40 mg kg^−1^. (1) Control group (saline solution injection) (2) Native-Fe_3_O_4_@MSN (3) DMPC-Fe_3_O_4_@MSN (4) PEG-Fe_3_O_4_@MSN. 

#### 4.5.2. Preliminary Toxicological Assessment

Four days after treatment, mice were sacrificed and organs, blood and urine were collected for histological analysis and biochemical assays. The blood samples with heparin were centrifuged at 1 300 rpm for 10 min. The plasma and urine were stored at −20 °C up to analysis. We measured plasma cytokines (TNF-α and IL-6) to assess the inflammatory reaction or systemic toxicity. Plasma TNF-α and IL-6 levels were quantified using commercial ELISA kits as described in the manufacturer’s protocol (R&D systems, Minneapolis, MN, USA). The evaluation of renal function was determined by measuring creatinine levels in plasma and in urine using colorimetric assay at 495 nm with alkaline picrate. Liver function was determined from alanine aminotransferase (ALT) activity. Plasma ALT activity was measured according to standard protocol (Infinity, Thermo Scientific, Waltham, MA, USA). Moreover, liver, spleen and kidney were fixed in 10% paraformaldehyde, embedded in paraffin and cut 5 µm tick sections in a microtome. Sections were mounted on glass slides. After staining with hematoxylin-eosin, the sections were examined and imaged under a light microscope. 

#### 4.5.3. In Vivo Distribution and Degradation of MSN

Inductively coupled plasma-mass spectrometry (ICP-MS) was used to quantify silicium distribution in the digested tissue samples (liver, kidney and spleen). 2–100 mg of biological samples (urine, blood, or lyophilized and crushed kidney, spleen or liver) were digested in HNO_3_ (1 mL, 67%) for 1 h, and then HCl (1 mL, 34%) and of H_2_O_2_ (0.5 mL, 30%) for 1 h. The digestion was completed by microwave in Teflon vials, by using UltraWAVE single reaction chamber (Milestone, Shelton, AL, USA). Silicium content was further analysed using a NexION™ 300 ICP-MS instrument (PerkinElmer, Waltham, MA, USA), from the analytical platform facilities of CEREGE (Aix en Provence, France). 

### 4.6. Statistical Analysis 

Statistical analysis was performed using the Student’s *t*-test to compare paired groups of data. A *p* value < 0.05 was considered as statistically significant.
